# Effectiveness of Training and Use of Novasil Binder in Mitigating Aflatoxins in Cow Milk Produced in Smallholder Farms in Urban and Periurban Areas of Kenya

**DOI:** 10.3390/toxins13040281

**Published:** 2021-04-15

**Authors:** Gladys Anyango, Irene Kagera, Florence Mutua, Peter Kahenya, Florence Kyallo, Pauline Andang’o, Delia Grace, Johanna F. Lindahl

**Affiliations:** 1 Department of Animal and Human Health, International Livestock Research Institute, Nairobi 00100, Kenya; gladysanyango0034@gmail.com (G.A.); i.kagera@cgiar.org (I.K.); F.mutua@cgiar.org (F.M.); d.randolph@cgiar.org (D.G.); 2 Department of Public Health, Maseno University, Kisumu 40100, Kenya; plandango@gmail.com; 3 Department of Human Nutrition Sciences, Jomo Kenyatta University of Agriculture and Technology, Nairobi 00200, Kenya; kyallofm@gmail.com; 4 Department of Food Science and Technology, Jomo Kenyatta University of Agriculture and Technology, Nairobi 00200, Kenya; pkahenya@gmail.com; 5 Natural Resources Institute, University of Greenwich, Central Avenue, Chatham Maritime ME4 4TB, UK; 6 Department of Clinical Sciences, Swedish University of Agricultural Sciences, 75007 Uppsala, Sweden; 7 Zoonosis Science Centre, Department of Medical Biochemistry and Microbiology, Uppsala University, 75123 Uppsala, Sweden

**Keywords:** mycotoxin binder, aflatoxin M1, smallholder dairy farmer, milk production, feed safety

## Abstract

Aflatoxins, which commonly contaminate animal feeds and human food, present a major public health challenge in sub-Saharan Africa. After ingestion by cows, aflatoxin B1 is metabolized to aflatoxin M1 (AFM1), some of which is excreted in milk. This study involved smallholder dairy farms in urban and periurban areas of Nairobi and Kisumu, Kenya. The objective was to determine the effectiveness of training and providing farmers with aflatoxin binder (NovaSil^®^) on AFM1 contamination in raw milk. A baseline survey was undertaken and 30 farmers whose milk had AFM1 levels above 20 ppt were randomly selected for inclusion in the study. Of these, 20 farmers were part of the intervention, and were given training on the usage of the NovaSil^®^ binder, while 10 served as a control group. All farmers were visited biweekly for three months for interviews and milk samples were collected to measure the AFM1 levels. The AFM1 levels were quantified by enzyme linked immunosorbent assay. The NovaSil^®^ binder significantly reduced AFM1 concentrations in the raw milk produced by the farmers in the intervention group over the duration of the study (*p* < 0.01). The control farms were more likely to have milk with AFM1 levels exceeding the regulatory limit of 50 ppt compared to the intervention farms (*p* < 0.001) (odds ratio = 6.5). The farmers in the intervention group perceived that there was an improvement in milk yield, and in cow health and appetite. These farmers also felt that the milk they sold, as well as the one they used at home, was safer. In conclusion, the use of binders by dairy farmers can be effective in reducing AFM1 in milk. Further research is needed to understand their effectiveness, especially when used in smallholder settings.

## 1. Introduction

The dairy subsector contributes significantly to health and economic wellbeing of communities in Kenya. Milk and milk products are important sources of nutrients, especially those often lacked by children and expectant mothers. Cow milk is the main type of milk used for human consumption and represents about 83% of the world milk production [1]. Milk in Kenya is mainly produced by smallholder dairy farmers [2]. 

Milk safety and quality are important in the realization of both health and economic outcomes. Milk contaminated above certain levels is not safe for human consumption and should be removed from the food chain. Contamination can be due to microbiological or chemical contaminants, such as mycotoxins. Mycotoxins are metabolites of fungi which cause negative health effects in exposed humans. These include aflatoxins, ochratoxins, citrinin, fumonisins, ergot, and patulin [3]. Aflatoxins, produced by fungi occurring naturally in the soil, are the most toxic mycotoxins, and are frequently found in cereals commonly consumed as human foods and used as animal feed, causing negative health effects and reduced productivity in livestock [4,5,6]. All aflatoxins have been classified as group 1 carcinogens [7]. Aflatoxin B1 (AFB1) is the most carcinogenic and after consumption by ruminants some is metabolized and excreted in the milk as aflatoxin M1 (AFM1). In addition to the huge economic losses from reduced livestock productivity [6] and discarded milk, AFM1 exposure from milk also contributes to increasing the incidence of liver cancer in Kenya and potentially also of stunting in children [6]; hence, actions to reduce its exposure are recommended. The high aflatoxin contamination of animal feed has also been reported in Kenya. In an earlier study that analyzed 412 samples, it was found that 86% of the samples were contaminated with aflatoxins (67% of which exceeded the FAO/ WHO limit) [8]. Another study reported high aflatoxin B1 (above 5 ppb) levels in 41 of the 74 feed samples analyzed [9]. Similarly, several recent studies reported AFM1 contamination in Kenyan milk [10,11,12]. Kang’ethe and Lang’a [8] detected AFM1 in 72% of milk samples analyzed; a contamination rate that translates to 3.7 billion liters of contaminated milk out of 5.2 billion produced. Samples collected from low-income areas of Nairobi were found to have detectable aflatoxin levels [12]. Previous studies reported that most of the milk sold in informal settlements in Nairobi was contaminated with AFM1, with levels above the recommended upper limits indicating an increased risk of exposure to consumers relying on this milk [11,13,14]. The survey in Kisumu County found 26.4% AFM1 prevalence in milk produced by smallholder dairy farmers [15] which was attributed to the poor feeding practices used by the farmers, such as the feeding of moldy feeds to cows. In the same county, processed milk and raw milk imported from neighboring counties, as well as the milk produced by the urban and periurban smallholder dairy farmers, were found to contain detectable levels of AFM1 [16]. 

Several mycotoxin-mitigation strategies in the milk value chain exist. Trials involving good agricultural practices, the proper storage of cereals, the decontamination of feed through dilution, and chemical treatment have been conducted, but with limited success [14,17]. No single approach on its own can address the problem of aflatoxins in the milk value chain, so there is a need for multiple measures at both pre- and postharvest levels. 

One strategy that can be used to control aflatoxins in milk is using mycotoxin binders. They are natural adsorbents with the ability to decrease bioavailability and reduce exposure to aflatoxins [18]. When used, and upon ingestion by an animal, the binders decontaminate mycotoxins in the feed by binding to them, thereby preventing their absorption from the digestive tract of the animal [19]. They are particularly recommended where feed is suspected to be contaminated with the aflatoxins and the likelihood of destroying it is very low, as is the case in many low- and middle-income countries. Several mycotoxin binders are sold on the market in Kenya. However, their effectiveness in preventing aflatoxin uptake varies with the type and amount added [20]. 

A good toxin binder may restore the nutritional values of aflatoxin-contaminated feed. Bentonite clays, which are rich in montmorillonite, have been effectively used in dairy cows to diminish the negative effects of aflatoxin exposure [18,21]. Montmorillonite rich calcium-bentonite has been shown to be effective in reducing aflatoxin biomarkers in serum and urine with negligible nutrient interactions in humans naturally exposed to aflatoxins via contaminated foods [22,23]. NovaSil^®^, a phyllosilicate clay rich in calcium montmorillonite, is considered a very effective mycotoxin binder due to its high binding capacity, high absorption efficacy, short activation time and ability to be used at a higher inclusion rate [24]. Apart from clays, other anti-mycotoxin additives have been tested, including buckthorn [25], and yeasts [18,26], with promising results in trials. Evidence is required to support the scaling up of mycotoxin binder usage by smallholder farmers in Kenya, and in similar settings in East Africa. The objective of our study was to determine the effectiveness of training smallholder dairy farmers on safe milk production and NovaSil^®^ binder use, with a focus on periurban farmers who are more likely to practice intensive farming given the limited land capacity and closeness to remunerative markets, providing motivation for the increased likelihood of feeding concentrate feeds and willingness to invest in inputs.

## 2. Results

### 2.1. Characteristics of Study Farms

The trial enrolled a total of 60 smallholder dairy farmers. Participant retention was 98%, with only one farmer leaving. Response rate was 96% and 99% in Kasarani and Kisumu counties, respectively, over all the six visits. The number of milking cows per household ranged from 1 to 18 cows. More male (60%) than female (40%) farmers participated in the study. Most farmers (63.4%) had attained secondary education, but no training on dairy production was reported (Table 1).

### 2.2. Milk Production 

Cows were milked twice a day. The farmers were smallholders, with an average baseline production of 24 (SD 26.6) L per farm, and a daily production of 34 (SD 32.2) and 14 (SD 13.9) L, for Kasarani and Kisumu counties, respectively. Milk production per farm ranged from 2 to 150 L per day. The overall mean milk production per cow was 7.1 (SD 3.9) L. The average milk production per cow for control and intervention groups, per study site, is summarized in Table 2. There were no significant differences (*p* > 0.05) between control and intervention farms within visit and site. An average of 21 L were sold per day at a mean of 67 Kenyan shillings per liter.

### 2.3. Farmers’ Perception on Use of Binder

All the intervention farmers reported using binders two times a day and this corresponded to the number of times they fed the cows with concentrate feeds each day. On each visit, all intervention farmers (100%) reported that it was easy to use the binder, that they knew how much binder to mix with feeds using the spoon provided by the project, and that the cows did not resist the binder-mixed feeds. Most farmers (99%) did not share their portions of binder with others (following the instruction they received during training). Compared to those in the control group, cows in intervention farms were reported to eat better (81% versus 37%), were perceived to be healthier (81% versus 40%) and had a better rating with regard to milk production (63% versus 33%) (Table 3).

### 2.4. Aflatoxin M1 Levels in Milk from the Study Farms

During the baseline there was no statistically significant difference in the milk production as well as AFM1 levels between the control and intervention farmers (*p* > 0.05). Overall, during the duration of the trial, mean levels of AFM1 in the control group increased compared to the means at baseline. The mean AFM1 levels in milk from farmers in the intervention group decreased compared to the baseline AFM1 levels. Farmers in the intervention group produced milk with lower levels of AFM1 compared to those in the control group *p* < 0.01 (Table 4). While the milk production decreased overall during the trial, there was no difference between intervention and control farms (*p* > 0.05) (Table 4 and Table 5). 

Over the duration of the trial, the mean AFM1 level was 78.9 ppt (SD 92.4, median 37.5 ppt) with a range from 1 to 538.9 ppt. With a mean of 73.2 ppt (SD 110.3, median 22.3 ppt) and a range from 1 to 538.9 ppt, AFM1 levels in more rural Kisumu were significantly lower than in more urban Kasarani (*p* < 0.05), which had a mean of 84.5 ppt (SD 69.9, median 68.6 ppt) and a range from 1 to 292.9 ppt. In Kisumu, AFM1 levels were significantly lower in the intervention (15.3 ppt) than in the control group (159.3 ppt) (*p* < 0.01). No significant difference in AFM1 levels between controls and intervention was observed in Kasarani (*p* = 0.86) (Table 5). 

The multivariable models showed a significant difference between intervention and control farms, with control farms having higher aflatoxin levels, and being significantly more likely to produce milk with AFM1 levels exceeding the regulatory limit of 50 ppt compared to intervention farms (*p* < 0.001) (OR = 6.5). Farms in Kasarani were also more likely to exceed the 50 ppt limit (OR 3.3, *p* = 0.007) (Table 6). The logistic regression model did not find any influence of average milk yield on aflatoxin levels, but this was found in the linear model; the log of aflatoxin levels increased by 0.08 for each liter of milk that was additionally produced by the cows. Regression models for price and milk production per cow revealed no impact of being part of the intervention or not.

## 3. Discussion

This study reports on the effects of an intervention which included training providing a commercial aflatoxin binder to smallholder farmers in Kenya to evaluate impact on the occurrence of AFM1 in milk from urban and periurban smallholder dairy farms, as well as the perceptions of farmers on the use and effects of the binder. Mean milk production in these smallholder farms was 24 L per farm and day. There was no significant better milk yield in the intervention group compared to that in the control group, even though most farmers perceived this. One of the effects of aflatoxin is reduced milk production in dairy animals [27], and these negative effects could have been mitigated in the intervention group by feeding the NovaSil^®^ binder, but the study was unable to show this. Feeding the cows binder was, however, shown to reduce the level of aflatoxin exposure, with no deleterious effect on milk production [28]. A decrease in milk yield was observed over time on both sites; however, this was not statistically significant. It is also likely that there were seasonal effects that affected the milk production in both sites, and both among control and intervention farms. 

This was a field trial where there was no control on the level of aflatoxins in the feed, and even though the farmers were instructed to feed first 1 teaspoon per 2 kg feed, and then 2 teaspoons, the researchers had no control of how much the cows were actually fed. This was by design, since the aim was to see the effects under normal farming conditions. It can be seen that the mean AFM1 levels in milk produced by the farmers in the intervention group in Kasarani reduced over time, with farmers having an average of 59.9 ppt during the final visit compared to 101.5 ppt at the third visit. AFM1 levels at visits 1, 2 and 3 seemingly increased under the low dose regime (1 teaspoon for 2 kg of feeds) of binder given to the cows. When increasing the dosage to 2 teaspoon per 2 kg tin of feeds, a decrease in AFM1 was observed in Kasarani, too. This was done because the AFB1 concentration in the feeds were likely higher than expected. AFM1 levels in milk in Kisumu reduced significantly during the four months of the trial. The AFM1 levels in intervention farms in Kisumu were consistently below the EU recommended limit of 50 ppt. This may be attributed to the farmers in Kisumu being more observant and treating the animals as advised, but it could also be due to real differences in contamination levels of the feed.

The overall average AFMI contamination levels was 78.9 ppt which was comparable to earlier results, with contamination levels of 84 ppt [29]. This study showed significant reduction of AFM1 levels in milk in the intervention farms, with the strongest effects found in Kisumu County. Similar results were observed in the United States, where dairy cows fed on AF-contaminated diet and NovaSil^®^ binder had significantly decreased AFM1 concentrations in their milk without affecting milk quality and composition [30]. In this study, the farmers did not report any abnormal signs upon feeding NovaSil^®^) binder to the cows. This was comparable with the findings of Maki et al. [30], where cows exhibited no abnormal behavior or clinical signs associated with aflatoxicosis. However, other studies have been conducted under controlled conditions, while this is the first study on the use of NovaSil^®^ binder by smallholder dairy farms in East Africa. This study has shown the potential of training and use of NovaSil^®^ binder in managing aflatoxin contamination problems along the dairy value chain.

## 4. Conclusions

The intervention effectively reduced AFM1 levels in milk and farmers were enabled to produce and sell milk with AFM1 levels below the EU recommended limit of 50ppt. There is a necessity for continued research on NovaSil^®^ effectiveness and cost-effectiveness in the smallholder dairy context, which predominates in Africa, in order to promote their appropriate use and understand their effect on the nutritional composition of milk, and their possible excretion in dung which many farmers use as manure. It is noted that the use of mycotoxin binders alone cannot solve the problem of aflatoxin contamination, and cannot replace good feed production, handling, and manufacturing practices, which are the primary control strategies.

## 5. Materials and Methods

Ethical review permit was obtained from the Institutional Research Ethics Committee of the International Livestock Research Institute, approval number ILRI-IREC 2017-10, approved on 31 March 2017.

### 5.1. Study Areas

**Figure 1:**
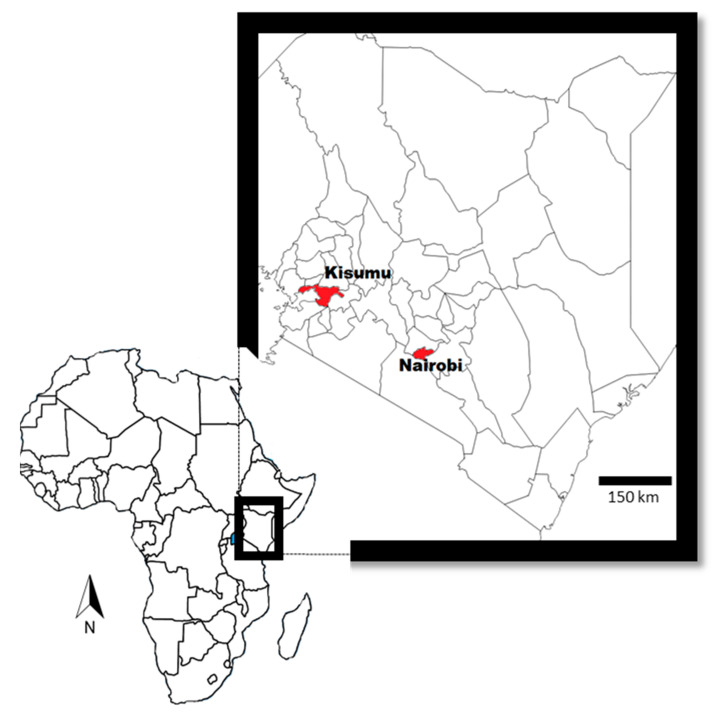
A map showing Kisumu and Nairobi counties.

The setting for the project is as described by Anyango et al. [15] and Kagera et al. [13]. Briefly, the study involved purposively selected urban and periurban areas of Nairobi and Kisumu counties (Figure 1). Both areas practice intensive smallholder dairy farming. In Nairobi, Kasarani subcounty was included. In Kisumu, which has a lower population, study farms were selected from five subcounties, namely: Nyando, Muhoroni, Kisumu Central, Kisumu West and Kisumu East.

### 5.2. Trial Design

An initial baseline survey involving 200 farmers preceded the trial [13,15]. The trial phase was carried out from July to October 2017. Farms whose milk had baseline AFM1 levels above 20 parts per trillion were considered in the NovaSil^®^ binder trial. Sample size was determined using the formula proposed by Metcalfe (2001); STATA sampsi 0.7 0.2, p(0.5) r(2) (assuming a reduction of positive farmers from 70 to 20%, using a power of 50% and a ratio of 2). This resulted in n = 60 farms (including 20 intervention and 10 control farmers in each site). At the start, the intervention group was given one day of training on improved dairy and food safety practices such as discarding of moldy feeds, proper ventilation of feeds while in storage, routine check up on feeds for dryness, mold growth, warmth, moisture, pests and animals, health risks of aflatoxin consumption, as well as mycotoxin binder usage, and each farmer was also provided with a package containing the NovaSil^®^ binder. A plastic tablespoon to aid measuring was also given. The recommended dosage rate was 1 teaspoon per 2 kg of feeds (estimated to equal 0.6% (6 g/kg) based on instructions given by the manufacturer). This dose was applied during the first half of the trial but was increased to 2 teaspoons per 2 kg (estimated to equal 1.2% (12 g/kg)). The change of protocol was because of suspected higher levels of aflatoxin contamination in the feed. Control farms were carefully selected to minimize the risk of spill-over of technology or information from farms receiving the intervention. Milk sampling and questionnaire administration was done every two weeks for three consecutive months (in total, six visits were conducted for each farmer); a longer gap was allowed during the 2017 election period for safety reasons. The questionnaire sought to understand: (i) how much binder farmers were adding to feeds; (ii) how much feed was mixed with the binder; (iii) whether the cows were eating: (iv) the challenges encountered while using the binder; (v) the farmers’ perception of drinking and selling milk from cows fed the binder-added feed; and (vi) how much farmers are willing to pay for the binders. A summary of the results was prepared and discussed within the project team before the next visit. Feedback was provided to the farmers in the subsequent visits, which also provided an opportunity to emphasize topics covered during the training and communicate the new advice on binder dosage. After the study, farmers in the control group received training on milk safety and binder use and were subsequently provided with NovaSil^®^ binder for three months. 

### 5.3. Collection of Milk Samples and Laboratory Analysis for Aflatoxin M1 (AFM1) in Milk

Farmers were alerted in advance about the next visit and asked to make sure they kept some of their milk for household consumption. Fresh raw bulk milk samples were then collected in sterile 50 mL flacon tubes and stored in cooler boxes then transported to ILRI laboratories where they were stored in a freezer at −3°C to −6°C awaiting AFM1 analysis. Milk samples were analyzed using commercial enzyme-linked immunoassay (ELISA) kit for AFM1 (Helica Biosystems, Inc., Santa Ana, CA 92704, USA, Catalog No. 961AFLM01M-96) according to the manufacturer’s instructions. The same approach had been used to determine AFM1 contamination levels at baseline [13,15]. The limit of quantification (LOQ) according to the manufacturer is 2 ng/kg. The ELISA has been evaluated previously and found to have good recovery and performance [31].

### 5.4. Data Analysis

Data were entered and cleaned in Microsoft excel 2013 (MS Excel^®^) and analyzed using SPSS (version 22) statistical package and STATA version 14.0. Results below the LOQ were substituted with half of the LOQ. Log transformation of AFM1 levels was done to attain a more normal distribution. Descriptive analyses for quantitative data included determination of measures of central tendency, including the mean (± standard deviation (SD)) and median. Categorical data were summarized using frequency tables, graphs, and trends. Inferential analyses included the use of Chi square statistics (to assess statistical associations) and Student’s t-test and ANOVA (to assess significance of differences in group means). All factors that could potentially affect AFM1 concentration in milk were included in the full model. Both multivariable linear and logistic regression were used to model the relationship between these factors and detection of AFM1 levels either as the log of the measured values, or as a binary variable with a cutoff of exceeding 50 ppt. A backward (manual) approach was used with Mixed and Melogit commands in STATA 14.2 (STATACorp, College Station, TX, USA), with repeated sampling accounted for by using clustering on farm level. Elimination of variables was done until only suspected confounders and those with significant (*p* < 0.05) associations remained in the model. Similar linear regression models were made using price and milk production per cow as outcomes. A statistical *p*-value of ≤ 0.05 was considered significant.

## Figures and Tables

**Table 1:** Household characteristics of study smallholder dairy farmers.

Characteristics	Kisumu	Kasarani	Total
	n (%)	n (%)	n (%)
	n = 30	n = 30	n = 60
Female	8 (26.7%) *	16 (53.3%)	24 (40%)
Male	22 (73.3%)	14 (46.7%)	36 (60%)
Mean age (years)	47.7	46.0	46.9
**Education level**			
Primary	8 (26.7%)	6 (20%)	14 (23.3%)
Secondary	13 (43.3%)	12 (40%)	25 (41.7%)
College/University	9 (30%)	12 (40%)	21 (35%)
Training on dairy feeding	11 (18.3%)	11 (18.3%)	22 (36.7%)

* Significant difference at *p* < 0.05.

**Table 2:** Average milk production (L ± standard deviation) per cow/day in the control and intervention groups, July–October 2017. Within the sites, there were no significant (*p* > 0.05) differences between control and intervention.

	Kasarani		Kisumu	
Time Point	Control	Intervention	Control	Intervention
Baseline	12.6 ± 6.4		7.0 ± 3.4	
1	9.5 ± 2.5	8.0 ± 4.9	5.5 ± 2.7	4.4 ± 1.9
2	8.5 ± 3.1	9.0 ± 4.5	4.9 ± 2.4	5.1 ± 2.6
3	8.1 ± 3.4	8.4 ± 4.8	5.1 ± 3.5	5.9 ± 2.6
4	9.0 ± 4.9	9.2 ± 5.6	5.7 ± 2.6	5.5 ± 2.8
5	8.6 ± 4.6	8.4 ± 4.1	5.7 ± 2.6	5.5 ± 2.8
6	8.7 ± 4.5	9.1 ± 4.1	5.7 ± 2.6	5.5 ± 2.8

**Table 3:** Perceptions of farmers on their cows during the trial period. Data are presented as absolute numbers and percentages of total respondents to the questions. Each farmer was visited six times.

		Intervention n (%)	Control n (%)
Feeding of cows ***	Better	185 (81.5%)	43 (37.0%)
	Same	36 (15.8%)	63 (54.7%)
	Worse	6 (2.6%)	9 (7.8%)
Health of the cows ***	Better	186 (81.5%)	47 (40.8%)
	Same	37 (16.2%)	62 (53.9%)
	Worse	5 (2.1%)	6 (5.2%)
Milk yield ***	Better	143 (63.5%)	37 (32.1%)
	Same	27 (12.0%)	43 (37.3%)
	Worse	55 (24.4%)	35 (30.4%)

*** *p*-value < 0.001 in Chi test.

**Table 4:** Average milk produced (L± standard deviation) and aflatoxin M1 (AFM1, ppt± standard deviation) levels in milk from by farmers in both study sites.

	Control Farms	Intervention Farms	*p*-Value
Mean AFM1 levels at baseline	79.8 ± 50.2	93.2 ± 63.0	0.51
Mean AFM1 over the duration of the trial	127.1 ± 119.0	54.4 ± 64.4	<0.001
Mean milk production at baseline	28.0 ± 22.9	39.1 ± 45.4	0.33
Mean milk production over the duration of the trial	20.8 ± 19.2	25.2 ± 29.4	0.15

**Table 5:** Mean milk production (L ± standard deviation) / farm and aflatoxin levels (ppt ± standard deviation) in milk.

	Kasarani						Kisumu					
	Control			Intervention			Control			Intervention		
Visit Number	N	Average Milk Yield (L)	AFM1 (ppt)	N	Average Milk Yield (L)	AFM1 (ppt)	N	Average Milk Yield (L)	AFM1 (ppt)	N	Average Milk Yield (L)	AFM1 (ppt)
Baseline	10	35.5 ± 30.8	0.87 ± 39.3	20	60.6 ± 55.6	132.2 ± 59.3	10	21.9 ± 12.6	68.6 ± 59.1	20	19.8 ± 20.4	54.1 ± 37.7
1	8	32.6 ± 26.5	98.3 ± 52.1	20	38.4 ± 42.2	82.1 ± 54.7	18	16.9 ± 16.2	164.2 ± 165.1	11	10.5 ± 4.9	37.7 ± 44.8 *
2	10	26.3 ± 25.7	75.1 ± 46.7	20	39.4 ± 36.4	98 ± 73.1	11	13.1 ± 8.3	156.3 ± 141.4	19	12.8 ± 13.7	31.5 ± 48.4 **
3	10	24.6 ± 26.2	81.9 ± 51.9	20	38.5 ± 38.2	81.5 ± 66.7	10	12.5 ± 8.2	136.1 ± 78.5	20	13.2 ± 16.4	22.2 ± 22.6 ***
4	9	26.6 ± 24.7	68.2 ± 81.2	20	39.4 ± 37.8	101.5 ± 83	10	15.7 ± 10.7	117.6 ± 89.7	20	13.9 ± 16.9	18.3 ± 21.0 ***
5	8	27.6 ± 25.8	97.5 ± 94.7	20	32.5 ± 30	88.1 ± 92.6	10	15.7 ± 10.7	180.1 ± 192.6	20	13.9 ± 16.9	16.3 ± 18.9 **
6	8	28.4 ± 26.2	81.7 ± 70.1	20	32.1 ± 25	59.9 ± 56.8	10	15.7 ± 10.7	201.4 ± 115.7	20	13.9 ± 16.9	10.1 ± 12 ***

*, **, ***: Significant difference within the site between control and intervention farms at *p* < 0.05, *p* < 0.01, and *p* < 0.001, respectively, using test on log (AFM1).

**Table 6:** Linear and logistic regression models for AFM1 levels in milk produced by farmers in the control and intervention group.

Predictor		Linear Model		Logistic Model	
		Increase in log (AFM1)	*p*-Value	Odds Ratio	*p*-Value
Control farm compared to intervention		1.09	< 0.001	6.52	< 0.001
Average yield L/cow		0.08	0.002	1.05	0.3
Kasarani compared to Kisumu		0.59	0.02	3.29	0.007
Visit compared to first visit	2	−0.21	0.3	0.6	0.3
	3	−0.19	0.4	0.78	0.6
	4	−0.36	0.1	0.52	0.2
	5	−57	0.01	0.47	0.1
	6	−0.67	0.002	0.34	0.02
		Estimate	Standard deviation	Estimate	Standard deviation
Random effect of farm		0.66	0.17	1.32	0.55
Residual AR (1)	Rho	0.038	0.072		
	variance	1.29	0.11		

## Data Availability

Data will be available upon request from the author.

## References

[1] McGuire S. (2015). FAO, IFAD, and WFP. The State of Food Insecurity in the World 2015: Meeting the 2015 International Hunger Targets: Taking Stock of Uneven Progress. Rome: FAO, 2015. Adv. Nutr..

[2] Oloo J. (2010). Food safety and quality management in Kenya: An overview of the roles played by various stakeholders. Afr. J. food Agric. Nutr. Dev..

[3] Wu F., Groopman J.D., Pestka J.J. (2014). Public health impacts of foodborne mycotoxins. Annu. Rev. Food Sci. Technol..

[4] Reddy K., Salleh B., Saad B., Abbas H., Abel C., Shier W. (2010). An overview of mycotoxin contamination in foods and its implications for human health. Toxin Rev..

[5] Stronider H., Azziz-Baumgatner E., Banziger M., Bhat R.V.R., Breiman R., Brune M.-N.M., Strosnider H., Azziz-Baumgartner E., Banziger M., Bhat R.V.R. (2006). Public Health Strategies for Reducing Aflatoxin Exposure in Developing Countries: A Workgroup Report. Environ. Health Perspect..

[6] Atherstone C., Grace D., Lindahl J.F., Kang’ethe E.K., Nelson F., Kang’ethe E.K., Nelson F. (2016). Assessing the impact of aflatoxin consumption on animal health and productivity. Afr. J. Food Agric. Nutr. Dev..

[7] IARC (2009). A review of human carcinogens: Chemical agents and related occupations. Lancet Oncol..

[8] Kang’ethe E.K., Lang’a K.A. (2009). Aflatoxin B1 and M1 contamination of animal feeds and milk from urban centers in Kenya. Afr. Health Sci..

[9] Makau C.M., Matofari J.W., Muliro P.S., Bebe B.O. (2016). Aflatoxin B1 and Deoxynivalenol contamination of dairy feeds and presence of Aflatoxin M1 contamination in milk from smallholder dairy systems in Nakuru, Kenya. Int. J. Food Contam..

[10] Senerwa D.M., Sirma A.J., Mtimet N., Kang’ethe E.K., Grace D., Lindahl J.F. (2016). Prevalence of aflatoxin in feeds and cow milk from five counties in Kenya. Afr. J. Food Agric. Nutr. Dev..

[11] Kirino Y., Makita K., Grace D., Lindahl J. (2016). Survey of informal milk retailers in Nairobi, Kenya and prevalence of aflatoxin M1 in marketed milk. Afr. J. Food Agric. Nutr. Dev..

[12] Kiarie G., Dominguez-Salas P., Kang’ethe S., Grace D., Lindahl J. (2016). Aflatoxin exposure among young children in urban low-income areas of Nairobi and association with child growth. Afr. J. Food Agric. Nutr. Dev..

[13] Kagera I., Kahenya P., Mutua F., Anyango G., Kyallo F., Grace D., Lindahl J. (2019). Status of aflatoxin contamination in cow milk produced in smallholder dairy farms in urban and peri-urban areas of Nairobi County: A case study of Kasarani sub county, Kenya. Infect. Ecol. Epidemiol..

[14] Kuboka M.M., Imungi J.K., Njue L., Mutua F., Grace D., Lindahl J.F. (2019). Occurrence of aflatoxin M1 in raw milk traded in peri-urban Nairobi, and the effect of boiling and fermentation. Infect. Ecol. Epidemiol..

[15] Anyango G., Mutua F., Kagera I., Andang’O P., Grace D., Lindahl J.F. (2018). A survey of aflatoxin M1 contamination in raw milk produced in urban and peri-urban areas of Kisumu County, Kenya. Infect. Ecol. Epidemiol..

[16] Obade M., Andang’o P., Obonyo C., Lusweti F. (2015). Exposure of children 4 to 6 months of age to aflatoxin in Kisumu County, Kenya. Afr. J. Food Agric. Nutr. Dev..

[17] Karlovsky P., Suman M., Berthiller F., De Meester J., Eisenbrand G., Perrin I., Oswald I.P., Speijers G., Chiodini A., Recker T. (2016). Impact of food processing and detoxification treatments on mycotoxin contamination. Mycotoxin Res..

[18] Diaz D.E., Hagler W.M., Blackwelder J.T., Eve J.A., Hopkins B.A., Anderson K.L., Jones F.T., Whitlow L.W. (2004). Aflatoxin Binders II: Reduction of aflatoxin M1 in milk by sequestering agents of cows consuming aflatoxin in feed. Mycopathologia.

[19] Whitlow L.W. Evaluation of Mycotoxin Binders. Proceedings of the 4th Mid-Atlantic Nutrition Conference.

[20] Mutua F., Lindahl J., Grace D. (2019). Availability and use of mycotoxin binders in selected urban and Peri-urban areas of Kenya. Food Secur..

[21] Kutz R.E., Sampson J.D., Pompeu L.B., Ledoux D.R., Spain J.N., Vázquez-Añón M., Rottinghaus G.E. (2009). Efficacy of Solis, NovasilPlus, and MTB-100 to reduce aflatoxin M1 levels in milk of early to mid lactation dairy cows fed aflatoxin Bl. J. Dairy Sci..

[22] Afriyie-Gyawu E., Wang Z., Ankrah N.-A., Xu L., Johnson N.M., Tang L., Guan H., Huebner H.J., Jolly P.E., Ellis W.O. (2008). NovaSil clay does not affect the concentrations of vitamins A and E and nutrient minerals in serum samples from Ghanaians at high risk for aflatoxicosis. Food Addit. Contam. Part A.

[23] Wang P., Afriyie-gyawu E., Tang Y., Johnson N.M., Xu L., Tang L., Huebner H.J., Ankrah N.-A., Ofori-adjei D., Ellis W. (2008). NovaSil clay intervention in Ghanaians at high risk for aflatoxicosis: II. Reduction in biomarkers of aflatoxin exposure in blood and urine. Food Addit. Contam. Part A.

[24] Marroquín-Cardona A., Deng Y., Taylor J.F., Hallmark C.T., Johnson N.M., Phillips T.D. (2009). Food Additives and Contaminants In vitro and in vivo characterization of mycotoxin-binding additives used for animal feeds in Mexico. Food Addit. Contam..

[25] Solcan C., Gogu M., Floristean V., Oprisan B., Solcan G. (2013). The hepatoprotective effect of sea buckthorn (Hippophae rhamnoides) berries on induced aflatoxin B1 poisoning in chickens. Poult. Sci..

[26] Weatherly M.E., Pate R.T., Rottinghaus G.E., Roberti Filho F.O., Cardoso F.C. (2018). Physiological responses to a yeast and clay-based adsorbent during an aflatoxin challenge in Holstein cows. Anim. Feed Sci. Technol..

[27] Applebaum R.S., Brackett R.E., Wiseman D.W., Marth E.H. (1982). Responses of dairy cows to dietary aflatoxin: Feed intake and yield, toxin content, and quality of milk of cows treated with pure and impure aflatoxin. J. Dairy Sci..

[28] Maki C.R., Thomas A.D., Elmore S.E., Romoser A.A., Harvey R.B., Ramirez-Ramirez H.A., Phillips T.D. (2016). Effects of calcium montmorillonite clay and aflatoxin exposure on dry matter intake, milk production, and milk composition. J. Dairy Sci..

[29] Langat G., Tetsuhiro M., Gonoi T., Matiru V., Bii C. (2016). Aflatoxin M1 Contamination of Milk and Its Products in Bomet County, Kenya. Adv. Microbiol. Kenya. Adv. Microbiol..

[30] Maki C.R., Monteiro A.P.A., Elmore S.E., Tao S., Bernard J.K., Harvey R.B., Romoser A.A., Phillips T.D. (2016). Calcium montmorillonite clay in dairy feed reduces aflatoxin concentrations in milk without interfering with milk quality, composition or yield. Anim. Feed Sci. Technol..

[31] Imtiaz N., Yunus A.W. (2019). Comparison of Some ELISA Kits for Aflatoxin M _1_ Quantification. J. AOAC Int..

